# Supplementary material for: Effects of Two Commercial Diets on Several Reproductive Parameters in Bitches: Note One—From Estrous Cycle to Parturition
Source: Animals (Basel). 2020 Dec 25;11(1):23. doi: 10.3390/ani11010023 (PMC7824412; doi:10.3390/ani11010023)
Supplement: Supplementary file 1 [file animals-11-00023-s001.pdf]

Article

Effects of Two Commercial Diets on Several Reproductive Parameters in Bitches: Note One—From Estrous Cycle to Parturition

Riccardo Orlandi [1,] Emanuela Vallesi [1], Alessandro Vastolo [2], Nadia Musco[2*], Serena Calabrò [2]Alessandro Troisi [3], Angela Polisca [4], Pietro Lombardi [2] and Monica I. Cutrignelli [2]

[1]Tyrus Veterinary Clinic, Via A. Bartocci 1/G, 05100, Terni, Italy; riccardo.orlandi83@hotmail.it (R.O.); manu0391@libero.it (E.V.)

[2]Department of Veterinary Medicine and Animal Production, University of Napoli Federico II, 80100, Napoli, Italy; alessandro.vastolo@unina.it (A.V.); nadia.musco@unina.it (N.M); serena.calabro@unina.it (S.C.); pietro.lombardi@unina.it (P.L.); monica.cutrignelli@unina.it (M.I.C.)

[3] School of Bioscience and Veterinary Medicine, University of Camerino, 62024, Matelica, Italy; alessandro.troisi@unicam.it (A.T.)

[4] Department of Veterinary Medicine, University of Perugia, 06124, Perugia, Italy; angela.polisca@unipg.it (A.P.)

* Correspondence: nadia.musco@unina.it (N.M.)

Received: date; Accepted: date; Published: date

Supplementary materials

Table S1. Bitches' serum biochemical parameters (m±sd) at recruitment.

**Hematic profile**	**BUN**	**Crea**	**Glu**	**TP**	**Alb**	**Bil T**	**AST**	**ALT**	**GGT**	[illegible]
Group	*mg/dl*			*g/dl*			*Ul/lt*			
							Treatment			
EX	44.0±4.3	1.55±0.13	131±0.5	7.15±0.07	4.12±0.10	0.47±0.10	31.5±1.1	29.5±1.2	3.50±0.27	[illegible]
CTR	74.0±8.3	1.20±0.19	134±0.7	7.45±0.09	4.27±0.15	0.41±0.13	26.8±1.4	30.5±1.6	3.80±0.42	[illegible]
							Size			
M	66.0±7.1	1.32±0.19	134±0.8	7.65±0.11	4.22±0.15	0.52±0.14	29.7±1.8	30.5±2.0	3.75±0.42	[illegible]
L	52.0±5.0	1.42±0.13	131±0.4	6.95±0.06	4.17±0.10	0.37±0.09	28.7±0.9	29.5±1.0	3.55±0.27	[illegible]

EX: experimental; CTR: control; M: medium; L: large; BUN: blood urea nitrogen; Crea: creatinine; Glu: glucose; TP: total proteins; Alb: albumins; Bil T: total bilirubine; AST: aspartateaminotransferase; ALT: alaninetransferasi; TRI: triglycerides; COL T: total cholesterol.

Table S2. Bitches mineral blood profile (m±sd) at recruitment.

**Mineral profile**	**Cl**	**Na**	**K**	**Na/K**	**Ca**	**Ca/P**	**P**
Group	*mEq/L*				*mg/dl*		
			Treatment				
EX	121±2	154±2	3.85±0.28	38.5±0.2	12.4±0.3	72.8±1.3	6.12±0.36
CTR	115±3	155±3	4.15±0.38	33.4±0.3	13.0±0.4	82.3±1.8	6.32±0.49
			Size				
M	118±3	155±3	3.85±0.41	36.2±0.4	13.0±0.5	89.8±2.1	7.35±0.53
L	118±2	154±2	4.15±0.26	35.8±0.2	12.4±0.3	65.3±1.1	5.10±0.34

EX: experimental; CTR: control; M: medium; L: large; Cl: chloride; Na: sodium; K: potassium; Ca: calcium; Ca/P: calcium/phosphorus; P: phosphorus.
